# Supplementary material for: Clinical Evaluation of Nerve Function in Electrical Accident Survivors with Persisting Neurosensory Symptoms
Source: Brain Sci. 2022 Sep 27;12(10):1301. doi: 10.3390/brainsci12101301 (PMC9599311; doi:10.3390/brainsci12101301)
Supplement: Supplementary file 1 [file brainsci-12-01301-s001.zip › brainsci-1898773-supplementary.pdf]

Supplementary Table S1: Individual results for patients: Mean values for warm and cold thermal change limit thresholds, in degrees Celsius; LEP latency and amplitude, in milliseconds and microvolts, respectively; and temperature roller detection, in reported detections. Solid circles indicate a test result that differs by two standard deviations, subnormal performance, from the reference value. Red dots indicate warm temperature and blue cold temperature deficiencies. For the temperature roller, any incorrect response is considered clinically relevant. Red or blue indicates the temperature of incorrect temperature roller response. No circle indicates no data available. 95% CI refers to a 95% confidence interval upper and lower range.

	Thermal threshold warm				Thermal threshold cold				LEP Latency				LEP Amplitude				Temp Roller			
	Hand		Foot		Hand		Foot		Hand		Foot		Hand		Foot		Hand		Foot	
ID	Left	Right	Left	Right	Left	Right	Left	Right	Left	Right	Left	Right	Left	Right	Left	Right	Left	Right	Left	Right
1	○	○	○	○	○	○	○	○	○	○	○	○	○	○	○	○	○	○	○	○
2	○	○	○	○	○	○	○	○	○	○	○	○	○	○	○	○	○	○	○	○
3	○	● (red)	● (red)	○	○	○	● (blue)	● (blue)	○	○	○	○	○	○	○	○	○	○	● (red) ● (blue)	● (red) ● (blue)
4									○	○	○	○	○	○	○	○	○	○	○	○
5									○	○	○	○	○	○	○	○	○	○	○	○
6									○	○	○	○	○	○	○	○	○	○	○	○
7	● (red)	● (red)	● (red)	● (red)	● (blue)	● (blue)	● (blue)	● (blue)	○	○	○	○	○	○	○	○	○	● (red)	○	○
8	○	● (red)	○	○	○	○	○	○	○	○	○	○	○	○	○	○	○	○	○	○
9	○	○	○	○	○	○	○	○	○	○	○	○	○	○	○	○	○	○	○	○
10	○	○	○	○	○	○	○	○	○	○	○	○	○	○	○	○	○	○	○	● (blue)
11	● (red)	● (red)	○	○	● (blue)	● (blue)	○	○	○	○	● (black)	○	○	○	○	○	○	○	○	● (blue)
12	○	○	○	○	○	○	○	○	○	○	○	○	○	○	○	○	○	○	● (blue)	○
13	○	○	○	○	○	○	○	○	○	○	○	○	○	○	○	○	○	○	○	○
14	○	○	○	○	○	○	○	○	○	○	○	○	○	○	○	○	○	○	○	○
15	○	○	○	○	● (blue)	● (blue)	○	○	○	○	○	○	○	○	○	○	○	○	○	○
16	○	○	○	○	○	○	○	○	○	○	○	○	○	○	○	○	○	○	○	○
17	○	○	○	○	○	○	○	○	○	○	○	○	○	○	○	○	○	○	○	○
18	● (red)	● (red)	● (red)	○	● (blue)	○	○	● (blue)	○	○	○	○	○	○	○	○	○	● (blue)	○	○
19	○	○	○	○	○	○	○	○	○		○	○	○		○	○	○	○	○	○
20	○	○	○	○	○	○	○	○	○	○	○	○	○	○	○	○	○	○	○	○
21	○	○	○	● (red)	○	○	○	○	○	○	○	○	○	○	○	○	○	○	○	○
22	● (red)	● (red)	○	○	○	● (blue)	○	○	○	○	○	○	○	○	○	○	○	○	○	○
23	○	○	○	○	○	○	○	○	○	○	○	○	○	○	○	○	○	○	○	○
24	○	○	○	○	○	○	○	○	○	○	○	○	○	○	○	○	○	○	○	○
**Mean**	**34.38**	**34.50**	**39.36**	**39.97**	**30.04**	**29.64**	**28.00**	**27.74**	**223.26**	**230.87**	**281.79**	**287.04**	**24.13**	**22.09**	**13.64**	**11.39**	100%	96%	94%	92%
**95%CI**	32.82 – 35.94	32.12 – 36.88	31.53 – 47.18	33.11 – 46.82	28.08 – 32.00	27.15 – 32.13	25.59 – 30.41	24.00 – 31.48	178.76– 267.76	183.32 – 278.42	229.7– 333.89	235.3 – 338.7	4.15 – 44.12	0.05 – 44.12	0 – 27.67	0.18– 22.60	Correct Response			

Supplementary Table S2: Individual results for patient's motor neurography, including motor latencies in milliseconds, motor amplitude in microvolts, motor conduction velocities (CV) in meters per second; and F-wave in milliseconds. Values that differs by 2 standard deviations, subnormal performance, from the reference value are marked with a solid dot. Ulnar below and ulnar above refer to the nerve recording either below or above the elbow. 95% CI refers to a 95% confidence interval upper and lower range.

	Motor Latency				Motor Amplitude				Motor CV						F-wave			
	Ulnar		Peroneal		Ulnar		Peroneal		Ulnar Below		Ulnar Above		Peroneal		Ulnar		Per.	
ID	Left	Right	Left	Right	Left	Right	Left	Right	Left	Right	Left	Right	Left	Right	Left	Right	Left	Right
1	○	○	○	○	○	○	○	○	●	○	○	●	○	●	○	○	○	○
2	○	○	○	○	○	○	○	○	○	○	○	○	○	○	○	○	○	○
3	○	○	○	○	○	○	○	○	○	○	○	○	○	○	○	○	○	○
4	○	○	○	○	○	○	○	○	○	○	○	○	○	○	○	○	○	○
5	○	○	○	○	○	○	○	○	○	○	○	○	○	○	○	○	○	○
6	○	○	○	○	○	○	○	○	○	○	○	○	○	○	○	○	○	○
7	○	○	○	○	○	○	○	○	○	○	○	○	○	○	○	○	○	○
8	○	○	○	○	○	○	○	○	○	○	○	○	○	○	○	○	○	○
9	○	○	○	○	○	○	○	○	○	○	●	○	○	○	○	○	○	○
10	●	○	○	○	○	○	○	○	○	○	○	○	○	○	●	○	○	○
11	○	○	○	○	○	○	○	○	○	○	○	○	○	○	○	○	○	●
12	○	○	○	○	○	○	○	○	○	○	○	○	○	○	○	○	○	○
13	○	○	○	○	○	○	○	○	○	○	○	○	○	○	○	○	○	○
14	○	○	○	○	○	○	○	○	●	○	○	○	○	○	○	○	○	○
15	○	○	○	○	○	○	○	○	○	○	○	○	○	○	○	○	○	○
16	○	○	○	○	○	○	○	○	○	○	○	○	○	○	○	○	○	○
17	○	○	○	○	○	○	○	○	○	○	○	○	○	○	○	○	○	○
18	○	○	○	○	○	○	○	○	○	○	○	○	○	○	○	○	○	○
19	○	○	○	○	○	○	○	○	○	○	○	○	○	○	○	○	○	○
20	○	○	○	○	○	○	○	○	●	○	●	●	○	○	○	○	○	○
21	○	○	○	○	○	○	○	○	○	○	○	○	○	○	○	○	○	○
22	○	○	○	○	○	○	○	○	○	○	○	○	○	○	○	○	○	○
23	○	○	○	○	○	○	○	○	○	○	○	○	○	○	○	○	○	○
24	○	○	○	○	○	○	○	○	○	○	○	○	○	○	○	○	○	○
**Mean**	**2.71**	**2.72**	**4.42**	**4.15**	**6.55**	**6.83**	**4.10**	**4.52**	**55.44**	**58.68**	**56.45**	**58.1**	**43.58**	**44.73**	**24.98**	**25.2**	**48.1**	**47.06**
**95%CI**	1.92 – 3.51	2.10 – 3.34	2.62 – 6.23	2.72 – 6.23	3.70 – 9.40	3.29 – 15.9	0 – 8.21	1.62 – 7.45	48.10– 62.78	52.86 – 64.46	58.88– 63.51	48.7 – 64.5	39.49 – 47.2	40.69 – 48.77	21.36 – 28.58	21.47 – 29.03	39.34 – 58.02	39.54 – 54.64

Supplementary Table S3: Individual results for patient's sensory neurography, including motor amplitude in microvolts, and motor conduction velocities (CV) in meters per second. Values that differs by 2 standard deviations, subnormal performance, from the reference value are marked with a solid dot. Ulnar palm, ulnar 3rd, and ulnar 4th refer to the nerve recording from reference to the palm, 4th (ring) finger, and 5th (little) finger, respectively. 95% CI refers to a 95% confidence interval upper and lower range.

	Sensory Amplitude								Sensory CV							
	Ulnar Palm		Ulnar 4th		Ulnar 5th		Sural		Ulnar Palm		Ulnar 4th		Ulnar 5th		Sural	
ID	Left	Right	Left	Right	Left	Right	Left	Right	Left	Right	Left	Right	Left	Right	Left	Right
1	○	●	○	○	○	○	○	○	○	○	○	○	○	○	○	●
2	○	○	○	○	○	○	○	○	●	●	○	○	○	○	○	○
3	●	●	○	○	○	○	○	○	○	○	○	○	○	○	○	○
4	●	○	○	○	○	○	○	○	○	○	○	○	○	○	○	○
5	○	○	○	○	○	○	○	○	○	○	○	○	○	○	○	○
6	○	○	○	○	○	○	○	○	○	○	○	○	○	○	○	○
7	○	○	○	○	○	○	○	○	○	○	○	○	○	○	○	○
8	○	○	○	○	○	○	○	○	○	○	○	○	○	○	○	○
9	○	○	○	○	○	○	○	○	○	○	○	○	○	○	○	○
10	○	○	○	○	○	○	○	○	●	●	●	●	●	○	○	○
11	○	○	○	○	○	○	○	○	●	○	○	○	○	○	○	○
12	○	○	○	○	○	○	○	○	○	○	○	○	○	○	○	○
13	○	●	○	○	○	○	○	○	○	○	○	○	○	○	○	○
14	○	○	○	○	○	○	○	○	○	○	○	○	○	○	○	○
15	○	○	○	○	○	○	○	○	○	○	○	○	○	○	○	○
16	○	○	○	○	○	○	○	○	○	○	○	○	○	○	○	○
17	○	○	○	○	○	○	○	○	○	○	○	○	○	○	○	○
18	●	○	○	○	○	○	○	○	○	○	○	●	○	○	○	○
19	○	○	○	○	○	○	○	○	○	○	○	○	○	○	○	○
20	○	○	○	○	○	○	○	○	●	○	○	○	○	○	○	○
21	○	○	○	○	○	○	○	○	○	○	○	○	○	○	○	○
22	○	○	○	○	○	○	○	○	○	○	○	○	○	○	○	○
23	○	○	○	○	○	○	○	○	○	○	○	○	○	○	○	○
24	○	○	○	○	○	○	○	○	○	○	○	○	○	○	○	○
**Mean**	**4.49**	**4.81**	**5.66**	**5.96**	**10.06**	**8.53**	**10.2**	**10.53**	**59.13**	**58.91**	**58.23**	**57.46**	**58.41**	**60.0**	**45.96**	**49.40**
**95%CI**	2.13 – 6.67	1.94 – 7.66	3.48 – 7.82	2.70 – 9.20	4.71 – 15.4	4.87 – 12.19	5.82 – 14.58	5.51 – 15.53	53.87 – 64.45	53.63 – 64.23	52.97 – 64.11	51.88 – 63.11	52.28– 62.53	54.08 – 65.96	40.32 – 51.56	43.56 – 55.43

Supplementary Table S4: Demographic and clinical characteristics of patients

Age in years	
Median (range)	41 (19–66)
Sex	
Male	19
Female	5
Latency from injury in years	
Mean (SD)	3.04 (1.57)
Median (range)	3 (1–6)
Worked as an electrician	
No	12
Yes	12
Exposure	
Low voltage (≤ 1000 V)	22
High voltage (> 1000 V)	2
Contact points	
Entry point on the upper extremity	24
Exit point on the upper extremity	15
Sought medical care	
No	2
Yes	22
If yes, stayed overnight	2
Days before returning to work	
Mean (SD)	1.08 (1.35)
Median (range)	1 (0–4)
